# Partnering for change: How a health authority, physicians, and communities work together to transform primary healthcare services

**DOI:** 10.1108/JHOM-02-2019-0032

**Published:** 2020-11-27

**Authors:** Martha L.P. MacLeod, Neil Hanlon, Trish Reay, David Snadden, Cathy Ulrich

**Affiliations:** 1 School of Nursing, University of Northern British Columbia, Prince George, Canada; 2 Department of Geography, University of Northern British Columbia, Prince George, Canada; 3 Department of Strategic Management and Organization, University of Alberta Alberta School of Business, Edmonton, Canada; 4 Northern Medical Program, University of British Columbia, Vancouver, Canada; 5 Northern Health Authority, Prince George, Canada

**Keywords:** Integration, Partnering, Primary care, Qualitative research, Organizational innovation, Rural health services

## Abstract

**Purpose:**

Despite many calls to strengthen connections between health systems and communities as a way to improve primary healthcare, little is known about how new collaborations can effectively alter service provision. The purpose of this paper is to explore how a health authority, municipal leaders and physicians worked together in the process of transforming primary healthcare.

**Design/methodology/approach:**

A longitudinal qualitative case study was conducted to explore the processes of change at the regional level and within seven communities across Northern British Columbia (BC), Canada. Over three years, 239 interviews were conducted with physicians, municipal leaders, health authority clinicians and leaders and other health and social service providers. Interviews and contextual documents were analyzed and interpreted to articulate how ongoing transformation has occurred.

**Findings:**

Four overall strategies with nine approaches were apparent. The strategies were partnering for innovation, keeping the focus on people in communities, taking advantage of opportunities for change and encouraging experimentation while managing risk. The strategies have bumped the existing system out of the status quo and are achieving transformation. Key components have been a commitment to a clear end-in-view, a focus on patients, families, and communities, and acting together over time.

**Originality/value:**

This study illuminates how partnering for primary healthcare transformation is messy and complicated but can create a foundation for whole system change.

## Background

Primary healthcare reform initiatives have been created to serve as the cornerstone of a transformed health system (Starfield *et al.*, 2005). Specific components include community involvement (Farmer and Nimegeer, 2014), new primary care organizations (Katz *et al.*, 2017), changes in service delivery approaches (Gilbert *et al.*, 2015), and interprofessional teams (Reay *et al.*, 2013). There remains, however, little evidence to guide the “how” of wide-scale transformation in primary healthcare, that is, how individuals and organizations within and across jurisdictions can work together to effect change. This is a critical oversight as effective primary healthcare relies on the interconnected contributions of individuals, groups and organizations that are not formally integrated through ownership or administrative hierarchy (Rummery, 2009).

One way to work together is to jointly undertake processes that generate collective effort and shared experience (i.e. partnering). The role of partnerships in effecting the integration of health and social care or health and public health have been well studied (e.g. Rummery, 2009; Hunter and Perkins, 2012). Less well examined, however, is partnering as a relational process. This study seeks to examine instances where collective actions are taken to transform primary healthcare within communities, but where formal partnerships between organizations or institutions are not in place. Research in such a setting may illuminate how relations amongst regional health care organizations, physicians, other providers and community interests can be activated to achieve healthcare innovation and improve the health of people within their communities.

In health system transformation initiatives that include primary care, collaboration and partnership have been primarily between physicians and those within the health system who provide community services (Denis *et al.*, 2011; British Columbia Ministry of Health, 2018). Despite the greater attention to the social determinants of health and health promotion (e.g. Suter *et al.*, 2009) as well as initiatives such as healthy communities (Hancock, 1993), local communities or municipalities seldom have been engaged, except with regard to specific health or social services they may provide (Stout *et al.*, 2017).

For the most part, healthcare transformation has been focused on making changes within the health system itself, even while recognizing that the health system exists as a complex adaptive system in an ever-changing context (Best *et al.*, 2012; Willis *et al.*, 2016). Despite the identification of principles for successful large system transformation that speaks to mechanisms of change within the health system (Best *et al.*, 2012) or key principles for integration (Suter *et al.*, 2009), more understanding is needed about how those mechanisms and principles are enacted. Such enactment includes the ongoing interplay of the context and social dynamics among key players that contribute to the advancement or delay of health and health service improvements (Stout *et al.*, 2017; Greenhalgh and Papoutsi, 2018). Examination of the actual ongoing processes of change is needed (Best *et al.*, 2012). As Langley *et al.* (2013) note, research that focuses on processes can capture the temporal nature of change, show how patterns of change are driven or are revised, and how interactions influence the dynamic nature of change across organizational levels and between organizations and their contexts. Close attention to how change happens over time both within and beyond the health system makes it possible to understand the complexities of change and the opportunities within it (Hunter *et al.*, 2015).

In Northern British Columbia (BC), Canada, there have been active efforts, especially since 2009, to build relationships among those involved in transforming primary healthcare services. In this environment, much has been learned about how key agents have forged productive and continuing relations, identified underlying issues, developed an agenda for change, and embarked on a long-term process of implementing patient-centered, organizational, and primary care practice transformations linked with health promotive changes in the communities.

## The health system context, partners and transformation objectives

### Geographical context: Northern British Columbia

**Figure 1:**
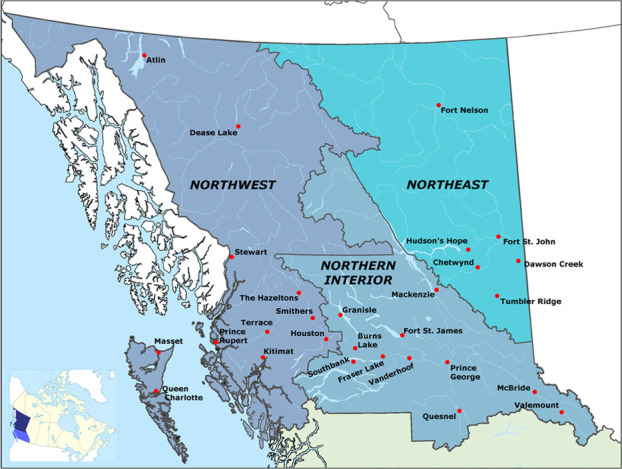
Map of Northern British Columbia

Northern BC encompasses a population of about 289,000 in an area of approximately 650,000 km^2^ that covers the northern two-thirds of the province. The region has the lowest population health status in the province (British Columbia Ministry of Health, 2014/2018). Approximately 17 percent of the population in Northern BC or 47,200 people are indigenous. The indigenous peoples are diverse: there are 54 first nations, 9 tribal councils and 17 distinct linguistic groups. Overall, the population of the region is younger than that of BC as a whole, with the population of persons 65 and over growing at twice the provincial rate. The region’s economic base relies mostly on resource extraction and primary processing, although the strength of community economies vary considerably and change over time. Thus, the demography, geography, and economy of Northern BC create particular challenges and demands for health and social services (Figure 1).

### Partners

#### Northern health

Northern Health is, geographically, one of the largest multi-site regionalized acute care and community healthcare systems in North America. It is one of five regional and one provincial health authorities in BC, all established in 2001. The First Nations Health Authority (FNHA), formed in 2012, oversees health services provided in First Nations communities throughout the province. Northern Health is responsible for acute care, long-term care, home and community care, public health, and mental health services throughout the region. Northern Health employs 7,000 people in 24 acute care facilities, 14 long-term care facilities, public health units and other specialized service teams organized within three health service delivery areas. Most physicians and many allied health professionals are not employees of NH. Northern Health contracts with many community-based support services and care providers, such as physiotherapists, youth mental health workers, and psychologists, who operate as private providers or within non-profit organizations.

#### Primary care physicians

Primary care physicians work throughout Northern BC. Physicians are independent practitioners traditionally remunerated directly from the BC MOH through fee-for-service arrangements. Most work in physician-owned or rented premises and within NH-owned hospital and emergency facilities. These arrangements have shifted in recent years to different types of compensation contracts and clinics owned by local communities or NH. Most, though not all primary care physicians, belong to one of six Divisions of Family Practice active in Northern BC (Divisions of Family Practice, 2018). The divisions were created to enable family physicians to work together to achieve common healthcare goals in collaboration with community and healthcare partners.

#### Municipalities

Municipalities are the most local form of elected government in Canada, incorporated in communities to organize and deliver basic public services (e.g. water treatment, sewerage, road maintenance, land use planning, fire protection, parks and recreation services). In Northern BC, there are a total of 31 municipalities, including 6 cities of 5,000 or more residents (of which only one has a population greater than 50,000), 14 district municipalities featuring towns of between 2,500 and 5,000 residents plus their surrounding rural areas, 1 town of approximately 5,000 people, and 10 villages of between 1,000 and 2,500 residents. Municipalities have traditionally played only very limited roles in health policy. More recently municipal governments are showing an interest in aligning community development activities to a population health outlook (e.g. active transportation, community gardens, social planning).

#### Healthcare transformation objectives: Northern Health’s system of services

The Northern Health Strategic Plans of 2009–2015 and 2016–2021, which were jointly developed through consultations with communities, physicians, and NH staff, have a clear vision and goals for fostering healthy, resilient communities, and a person- and family-centered approach to health services (Northern Health, 2018a). These strategic plans have shaped how the NH Board of Governors has actively built governance and service delivery relationships with northern communities and Divisions of Family Practice in order to deliver their mandate from government in the context of northern BC.

While the overall goal and principles of a transformed health system have been consistent across the region, different approaches to service reconfigurations and realignments have been undertaken in each community, based on the particular population health needs, medical practices, community priorities, and healthcare provider availability. A separate but interlinked process has been underway with the FNHA and First Nations communities to create a health delivery system that is experienced as being culturally safe (Greenwood, 2019).

**Figure 2:**
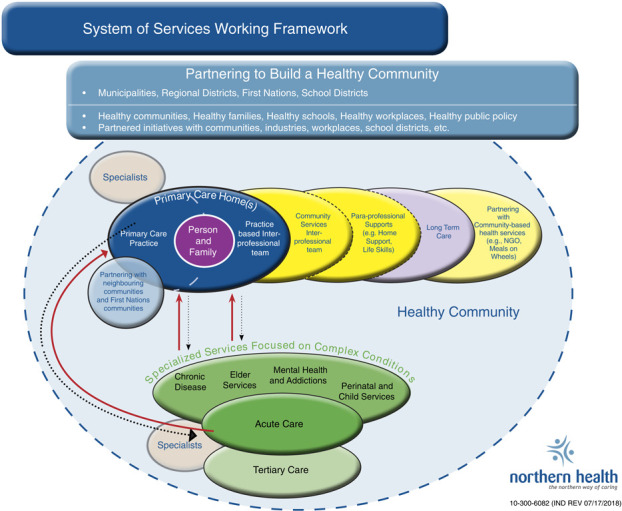
Place system of services working framework here

The NH System of Services Working Framework, first articulated in 2012 and subsequently revised (Figure 2), depicts the reoriented healthcare system within which primary care providers (physicians and nurse practitioners) and specialist physicians work with integrated primary healthcare teams. It outlines how the other specialized services, acute care, and long-term care services interconnect within the health system and with other organizations, health and social service providers, and communities to provide patient, family and community-based services.

The following principles underpin the working framework:

- working from a community-informed, collective strategic vision;
- providing patient and family-centered services within the context of the community;
- engendering a collaborative approach: creating trusting relationships and partnerships for planning and services;
- recognizing and supporting the generalist nature of rural communities and rural professional practice;
- partnering among physicians, communities and NH;
- supporting shared care between primary care providers and specialists;
- supporting interprofessional teams;
- providing care and services at a distance;
- fostering community-based change; and
- changing NH processes and structures to provide equitable services across the region.

The system of services is shaped around Primary Care Homes (Northern Health, 2018b), where persons and families can establish a long-term relationship with a primary care provider and an interprofessional team to receive coordinated, longitudinal care with linkages to specialist physicians and specialist teams as needed. Primary Care Homes are embedded in a healthy community and to that end, health promotion, illness prevention, and a population orientation are integral to their work. Primary Care Homes extend the current conception of the Patient Medical Home (Katz *et al.*, 2017) and are consistent with the current directions of the BC MOH’s efforts to promote primary care system change (British Columbia Ministry of Health, 2018).

The system transformation has seen progress in several areas: the creation of functional, long-term relationships among communities, NH and physicians that has allowed for system change; the creation of Primary Care Homes with integrated, NH-based interprofessional teams that interconnect with specialist and broader community-based health and social services; and the establishment of partnered, municipally led community initiatives, along with the joint recruitment and retention of primary care providers. Such progress has provided the foundation for further primary healthcare transformation.

## Methods

The study focused on how the changes were undertaken, that is, how relationships were established and evolved, and how NH, physicians and municipalities (communities) worked together to change health services over time and within specific contexts and places. A longitudinal case study (Stake, 1995, 2005) was undertaken using an interpretive qualitative approach. The case was bounded by the geography of NH and the time of organizational changes from 2010 to the end of data collection in 2015. Ethical approval was received from the researchers’ universities and NH’s Research Review Committee.

### Study participants

Study participants were recruited in seven communities that were identified by the NH knowledge user partners. The largest community in NH was Prince George, a city with a population greater than 70,000. The other six communities ranged in population size from approximately 1,000 (two communities), fewer than 5,000 (two communities) and between 10,000 and 20,000 (two communities). The communities varied in geographic, economic, cultural and social contexts; all were located more than 400 km from a metropolitan center of 250,000 residents or more.

Key informants were recruited through both purposive and snowball techniques. The initial list of potential participants was created through discussions with NH, as well as through an analysis of planning documents and evaluation data. Those who participated were asked to provide the names of additional individuals. Community participants included physicians, nurse practitioners, nurses, other allied health professionals, managers, municipal leaders (mayors and councilors) and community members (volunteers or municipal employees) identified as playing important roles in facilitating primary healthcare change and innovation. Regional participants included NH Executive, Board members, administrators and directors of relevant programs. A total of 122 participants provided a total of 239 interviews over three rounds of interviews (see Table I). The intent was for 7–8 participants per community each year. In Years 2 and 3, where possible, the same persons were interviewed, but some were replaced due to participants leaving the communities. Consent was received in writing prior to the first interview and by verbal consent afterwards.

### Data collection and analysis

In-depth, semi-structured interviews were conducted in-person by the first author (regional interviews) and a trained research assistant (community interviews). Interviews focused on opportunities for making changes to improve the health of the persons in the community; innovations that were attempted; key individuals involved; how trust and tensions were experienced. See the Appendix for the interview guide. Interviews lasted from 60 to 90 min, were digitally recorded and transcribed verbatim. Field notes and minutes from networking meetings, planning functions and public meetings, as well as policy documents and reports served as background to the interviews and have been reported on elsewhere (Hanlon *et al.*, 2019a).

All interview data were systematically analyzed through a multi-stage iterative process with the use of NVivo 10 (QSR International) for initial coding. Through repeated and sustained readings of the parts and the whole of the transcripts, the researchers considered similarities and differences within and across interviews, ideas that stood out, and statements that called previous understandings into question (Moules *et al.*, 2015). The first step was to review data from the participants in each community and regionally in each year, followed by reviewing data from similar types of participants across communities and years. For example, all interviews with physicians were explored for processes of engagement (Snadden *et al.*, 2019). Patterns in the data that expressed how change occurred through partnering were identified. These themes were further refined through discussion among the research team with a process of cross-checking the investigative team’s interpretations of the data for logical consistency and wherever feasible, empirical support from other sources of data. The goal was to keep the context as fulsome as possible (Langley, 1999) in order to understand the interconnections of process within its context. Rigor was achieved in several ways (Patton, 2014): using multiple sources of data, engaging a range of participants, clarifying participants’ understanding through multiple interviews, and receiving feedback on the themes and interpretations from participants and others in NH as well as at meetings of community and municipal leaders over the course of the study.

## Findings: transforming a system of care through partnering

Comparing initial and end-of-research interviews, participants reported varying degrees of change in primary healthcare. Early transformations were evident in changes in service provision, involvement of a wider range of healthcare professionals in care, and willingness of partners to design and implement changes in service provision. For example, a revised process for perinatal care was created, which resulted in 100 percent (compared with a previous 50 percent) of women being seen in the first trimester by public health nurses. The result was increased comprehensiveness and continuity of care, and better service provision for women with financial, social, alcohol, and smoking concerns:

[We made major changes.] When a pregnant woman phoned the physician’s office to make that [first] appointment, the physician’s office would [immediately] book their appointment with public health nurses. So non-NH staff was booking for NH’s Public Health [which was revolutionary] […]. And then when they left the public health nurse, our clerk set up their appointment with the physician’s office for their first prenatal assessment […] usually they see them a week to two weeks later […].

(Nursing Coordinator, Year 3)

A change in process was also created to improve access for patients who needed mental health services. As one physician noted in Year 3, co-charting with community-based Mental Health services (mental health nurses, care workers, financial services workers) was an important innovation that allowed for more comprehensive care, easier referrals, “better patient handoffs,” and seamless care. Such changes were beginning to be experienced across northern BC by the end of the data collection period.

The four strategies for achieving change were: partnering for innovation, keeping the focus on people in communities, taking advantage of opportunities for change, and encouraging experimentation while managing risk. While proceeding separately, these strategies were in continual interaction.

### Partnering for innovation

Three key ways in which the NH, the municipal leaders and the physicians went about partnering for innovation were to engage in purposeful conversations, respect multiple views, and pay careful attention to language.

#### Engaging in purposeful conversations

Establishing and continuing purposeful conversations have been viewed as an important step in collaboration. Regular meetings and joint planning sessions have led to common understandings, intersecting initiatives, and two-way alignment of service provision goals. The NH Board has held open roundtable sessions with communities several times a year; it has met biennially with the municipal leaders across Northern BC, including leaders of First Nations communities, around building healthy communities and working in partnership. Jointly with the communities, NH scanned community health indicators, mapping current health and health-related services in the community, and identifying key health issue(s) for action. After jointly identifying needs and resources, communities have initiated upstream primary healthcare initiatives, with seed funding from NH. For example, one small community began a bicycle-friendly initiative; another collaborated with its health center to create walking paths – strategies all focused on improving health and wellbeing.

This work has meant reconciling differing agendas. As a Northern Health Executive said in Year 1:

We have to align with them to build this partnership. You don’t want to graft them onto what we do. And we’ve got to build this partnership very respectful for who they are and where they want to go. And in some cases, that’s working well and in other cases we’re rejigging.

And one municipal leader noted in Year 3, “Our relationship […] has been superb. They’re really looking at ways to problem solve as opposed to not collaborating. So I value that relationship, because it feels equitable.”

Part of the early efforts focused on identifying the “right people.” Board members took time to engage mayors and municipal councilors; at the local level, community nurses convinced physicians and local recreation leaders to participate:

Sometimes we get the people right, and sometimes we don’t […]. But I find that once you get them in the room and you have some kind of a shared vision, and you just kind of be open to ideas, that things work out […]. [what’s important is] the frontline staff -- because they are the ones touching and seeing the clients and they see the processes where they fall apart and where they work.

(Community Manager, Year 3)

As a result, a shared sense of accountability has arisen. As one municipal leader in Year 2 said:

If it is not our responsibility, are we facilitating any action to try and make sure that somebody becomes responsible? […] It is having conversations and forming partnerships with organizations like NH and saying how do we work together?

By engaging those who can implement change due to their positional or informal authority, working relationships have changed in ways that support new services.

#### Respecting multiple views

Traditionally, physicians and health authorities have had difficulties in developing trusting relationships. The newly formed Divisions of Family Practice have facilitated joint planning with physicians at both the governance and local community levels. This was a critical step because it has led to new ways of allocating resources and transformed health services. As one physician said:

[…] it’s become a more durable relationship, and a more trustworthy relationship over the last few years. […] Northern Health has really embraced the same concept of Primary Care Homes that we believe is the important part. We came from different directions to hold that value at the center of primary care. […] It will be a better experience for patients and in turn, their health is going to be better because of what the health authority is committed to. And I think our members are coming to better understand their role in primary care with better clarity.

(Physician, Year 1)

The work with the Divisions has allowed physicians and NH to implement a new shared vision of a re-configured system to better serve patients.

#### Paying careful attention to language

Using language in ways that facilitate change is the third approach. Achieving a balance between change initiated regionally and that initiated within communities has not been easy. Northern Health executive team members, in particular, have noted the need to be acutely attuned to how language reflects intention and action:

Language is very important in this work that we’re doing, it’s huge […] So what do I hear now? […] ‘I can’t say roll out, […] I can’t say drive out’. […] it’s implement, it’s support.[…] And it’s about the language of community, the strength of community, how it gets supported […] because people take our words to heart and they believe them and they repeat them. So we have to be really mindful of our language because language is culture […]. You’ll hear people correcting themselves now.

(NH Executive, Year 1)

This attention to language has been identified as critical in influencing the development of respectful relationships and the achievement of creative, contextualized and supportive action.

### Keeping the focus on people in communities

The second strategy was to purposefully keep the focus on people in the communities. This happened in two ways: putting person and community-centered structures and processes into place, and building in flexibility to adapt to local circumstances. These approaches began with tracing patient journeys through the healthcare system. Clinicians and managers alike were surprised to find repeated instances where efforts were duplicated, where patients or family members were required to tell and re-tell their stories, where patients were repeatedly assessed with multiple assessment forms, and where co-location of services did not translate into coordination or continuity of care. These findings revealed the importance and urgency of finding new ways to provide services.

#### Putting person and community-centered structures and processes into place

Health services are all too often shaped by policy and professional practice expectations determined in isolation from patients and families. An example is documentation and reporting systems. Although home and community care, mental health and addictions, and public health have been the responsibility of the Health Authorities since 2001, the echoes of provincial directions are evident in provincial documentation and reporting requirements. Such requirements have not yet been harmonized with primary care physicians’ medical records. The lack of harmonized electronic medical records (EMRs) makes it challenging for an interprofessional team to focus on the patient and family at the local level while meeting regional and provincial informational needs. As one regional leader noted, the data system required for provincial and national public health reporting presented a challenge to keep the focus on the person. As he said:

[The system] was built for vertical health care delivery in Public Health but doesn’t [facilitate]a horizontal service at all, because to provide good health care, you need to have all those teams working in an integrated sense. [We’ve created] all these information silos that […] will not help the person actually delivering health care. It’ll help at the regional level […] but it’ll just be cumbersome. When I’ve talked to nurses in [small towns] about their frustrations, they say there are so many systems to log into and they spend so much time documenting that it becomes a struggle for them.

(Regional Director, Year 3)

To help integrate services between primary care physicians and primary healthcare teams, NH adopted a community EMR that interfaces with the EMR used by most primary care physicians in Northern BC. It also interfaces with the hospital systems and the provincial documentation system for public health. The goal was to enable the creation of a shared plan of care between patients and their families, the primary care providers (physicians and nurse practitioners), and the primary healthcare team. Although integrated EMR systems are not yet fully in place, NH clinicians and physicians are now able to better focus on caring for persons and their families locally, while meeting regional and provincial reporting requirements.

Health action committees in multiple communities have incorporated feedback from patient and family experiences with the health and social systems as part of efforts to develop new structures for joint planning and action. One such committee prioritized action on addiction prevention and services through partnerships among the Salvation Army, RCMP, the municipality, the school district, local First Nations, local physicians, NH and community health agencies. The committee developed an action plan that was grounded in respect for each organization’s role in meeting community needs. As one community leader said about the community action process, “It really is about ‘what’s your role in this and how can we work together?’”

#### Building in the flexibility to adapt to local circumstances

Implementing new approaches to primary healthcare has happened differently across communities to accommodate variation in medical practice arrangements and payment schemes. Thus, the tools, incentives and levers for engagement among NH, communities, and physicians must be adaptable. As an NH Executive member (Year 1) stated:

[…] it isn’t […]. about dictating out from the centre, we’ve got to get out there and talk to them […]. We’re coming out to facilitate a discussion so you can get the same ‘Ah hahs’ that we’ve had. Or different ones. And so that takes time, this is a big region with a lot of communities and you can’t sort of say, ‘Well they had that discussion in [community A], talk to them. They gotta have that discussion, every one of them has to have that discussion if they’re going to move it forward substantially.

In 2014, NH’s middle managers with responsibility for all health services in a particular community, including acute care, took on the responsibility of integrating primary healthcare fully into the system. New collaborations with “representatives from across the whole social services spectrum in the community right from the RCMP to the college, to [primary care providers] […]” (Middle Manager, Year 3) allowed for adapting service delivery. As a municipal leader in Year 3 said:

As long as Northern Health continues to consult and we know when they’re doing some of these things that they’ve done, like Men’s Health and the cancer piece that they did, those kinds of things where they come in and they talk to the community instead of just saying, “here it is,” plop, makes quite a difference to people. Cause then they feel they’re part of the process.

Locally based planning has now become a cornerstone in the process of implementing primary healthcare initiatives.

### Taking advantage of opportunities

Northern Health has consistently been “strategic, yet opportunistic,” building on a clear “end-in-view” (Dewey, 1922) of improved health for the northern population. Two approaches to taking advantage of opportunities were apparent: watchful waiting, and taking advantage of crises to implement the integration of services.

#### Watchful waiting

The NH Executive team has been determined to set expectations, and then support change within communities and within NH itself. As one NH Executive member noted, local changes began with “softening up the system”:

There is a whole different [outlook] […] managers problem-solve [a patient-centred] scenario in a way that they wouldn’t have done in the past. […] they use the word “soften” and they almost mean it literally. It’s like things are getting more flexible […], it’s a little more fluid, people aren’t so rigid, they’re willing to talk about a solution.

(NH Executive, Year 3)

Intentional and opportunistic planning efforts were implemented, first in prototype communities, and then across the region. Efforts were not designed to fix the existing system, but rather to create a new system through partnering among NH, physicians, and communities. Regional processes enabled joint efforts to implement changes across communities:

[We] share process maps across the whole organization. So it’s a good way to share the process that’s expected and refine it. […] we can share our learnings between communities and between the different caregivers […].

(Regional Director, Year 3)

Change processes were also opportunistic. Meetings amongst physicians, practice support staff, and NH staff allowed the spread of innovations to begin early. For example, the deployment of some information technology staff to work with physicians, nurses, and others in small communities increased EMR use. At the sub regional level, front line managers engaged with providers and community leaders in smaller communities:

[I’ve started] to build capacity with other people in other communities and I’ve done some work with [district municipality], I’m doing some work with [village], so again moving outside of the boundaries of [small city] […].

(NH Community Manager, Year 3)

Watchful waiting of emerging change by the NH Executive was necessary but not sufficient. Deliberate changes in processes and structures took place when Northern Health undertook a complex workforce transition process between 2014 and 2016, together with labor unions.

The workforce transition process reorganized staff from the “silos” of community health services (mental health and addictions, public health, and home and community care) into primary healthcare teams, linked to Primary Care Homes, and created the new role of Primary Care Nurse. Links between specialist services and Primary Care Homes were designed to keep services as close to home as possible. For example, generalist (Primary Care Home based) mental health services and specialist mental health services were delineated and referral/feedback processes articulated. Despite these necessary, deliberate processes, watchful waiting on the part of the NH leaders allowed for support of local initiatives followed by decisions about specific ways to integrate services with Primary Care Homes, how interprofessional teams would manage workload, and when specialized services were needed.

#### Responding to crises

In at least two communities, when a shortage of physicians became acute, NH, the physicians, and the communities used the crises as opportunities for change. The process was not straightforward, nor without tensions. Rather than responding to the challenge with a more traditional response (e.g. temporary locums; conventional recruitment efforts), the physicians, community leaders; and NH sought to make significant changes in the system while maintaining functional coverage for medical care.

In one small city, the BC Ministry of Health, in collaboration with physicians and NH introduced an innovative physician compensation model that enabled physician retention. At the same time, an interprofessional perinatal service was quickly created to improve patient-centered services for childbearing families, along with the creation of an unattached patient clinic involving nurse practitioners.

In a district municipality, the community created a primary care society to address problematic physician turnover. With the support of NH and engagement of physicians, a community facility was renovated to allow for co-location of providers and the interprofessional team. This engagement of all the partners has resulted in a stable team of primary care providers, who also provide medical and nursing education.

In both communities, the hard work of building and maintaining trusting relationships among NH, community leaders, and physicians was necessary to address the crisis. The physicians and NH acknowledged the tensions between them, confirmed their joint goals, and took concrete steps towards working as partners. As a result, there was rapid mobilization of new services. There’s “a lot of anxiety but there’s a feeling from the leaders of the physician group that we’re going to sort it out together” (NH Executive, Year 3).

It has not been easy to determine the optimum pace of change or to support community-based evolutionary work while still meeting Ministry of Health needs for standard, documented, planned, structured processes with clear lines of action and accountabilities.

### Encouraging experimentation while managing risk

In their efforts to transform the health system, the partners have all experimented by creating the space for communities and services to try new initiatives. At the same time, those initiating change have experienced tensions and counter-pressures that are somewhat commonplace in healthcare, such as competing and contradictory priorities (e.g. central vs local), and various forms of organizational inertia. Finding a balance between taking and managing risk is challenging, and especially so in the contexts of provincial directives and of partnering.

#### Creating space to try new initiatives and push existing boundaries

Innovations in primary healthcare have occurred in different ways in different communities while ensuring a consistent regional direction in concert with provincial directions. As a Regional Director noted:

[…] the other health authorities, I see them bumping up against other parameters that are around them, set out by their own health authority. That doesn’t happen with this [primary care transformation] work particularly because the strategic plan is guiding us to move forward in this way.

(Regional Director, Year 2)

The delivery of multidisciplinary specialty services through the Primary Care Home has been made possible by the reconfiguration of NH’s community health service providers into interprofessional primary healthcare teams, working with physicians and nurse practitioners. This reconfiguration has created space for new initiatives. For example, palliative care services are now provided throughout the region by primary care providers and the interprofessional teams, with 24/7 assistance from a small, specialist palliative care support team of physicians, nurses, and pharmacists who, in addition to teleconsultation, provide on the ground clinical problem-solving and education.

Although there has been progress, this is an unfinished, dynamic area in which tensions between the partners surface on occasion and are worked through. The partners have learned that where decision making and planning has been shared, each partner has taken calculated risks, including the risk of losing control. When the integrity of their shared vision and their relationships have been maintained, however, they have been able to create innovative, responsive, patient and family-centered services across Northern BC.

#### Managing organizational risk

Throughout the primary healthcare transformation process, different parts of the system have been ready to move at different times; change has occurred at different paces. This variability has threatened the change itself as well as the partners. Some physicians and communities have felt as though they were held back because regional systems were not ready to support local initiatives. For example, adopting the community EMR at the same time as creating new approaches and systems has required adjustments to manage the risks of mis-timing and mis-communication:

[The system] is only possible because of the fact we have leadership engagement and endorsement. And because we have a strategy around primary healthcare integration. This isn’t just about putting in a [EMR] tool. More than any project we’ve ever done in Northern Health, we have the work flow and how we do the service part going on in parallel with how we’re working and building the tool. And in both parallel efforts, there are a lot of unknowns. We’re working through this as best we can and then as we hit roadblocks, like how do we deal with [provincial system], we have absolute support.

(Regional Director, Year 3)

One of the largest risks was the workforce transition plan. The plan addressed issues such as harmonizing positions within different unions; workers who once were in different teams were now working within the same interprofessional team. As one NH Community Manager noted in Year 3, “We still have lots of union issues to sort through because the collective agreements are based on an old world that made sense at the time but doesn’t make sense anymore […].” Importantly, the creation of Primary Care Nurse positions prompted much change. The decision to name the role, Primary Care Nurse, was deliberate, as it signaled the substantive change in expected ways of working. For many nurses, becoming a Primary Care Nurse meant a new world of work, “We’re being advised that they want us to approach our nursing practice as generalists and not specialists” (NH Nurse, Year 3). The risks of alienating a large part of the nursing workforce have been ameliorated through consistent communication, clarification of competencies and expectations, and provision of supportive informal and formal education.

The goal of interconnecting the interprofessional team around a Primary Care Home could only be met with the transition of the workforce into roles that would work within interprofessional teams, work well with physicians and suit the requirements of particular communities. In making this change, the NH Executive and Board made decisions that balanced the risks of inertia to the organization and its partners vs the risks of creating new roles and ways of working. Making such decisions has meant weathering considerable controversy.

## Discussion

The goal of this study was to explore how a health authority, municipalities and physicians worked together in a process designed to transform primary healthcare in order to improve the health of the population. A critical overarching requirement was to find new ways of providing services rather than rearranging parts of the existing system. Four strategies with nine approaches were identified. Instead of setting strategies with fixed dimensions, the processes of transformation could more aptly be characterized as an ongoing, iterative process in which partnering plays a major role. Northern Health, municipal leaders, and physicians have been taking a deliberate, thoughtful approach to change, keeping the end-in-view, addressing as many contingencies as possible, but not letting the need for certainty retard the progress of change. Change has been both strategic and opportunistic. All partners are changing individually as they work together.

Partnering in Northern BC has not been of formal, institutional partnerships, but rather as a series of interactions between organizations and individuals that has occurred simultaneously at multiple levels. A key strategy is mobilizing the “right people” and engaging in purposeful collaborations, characterized by respect. Showing up at meetings on each other’s home ground, prepared to work together, and following up with action, has facilitated the development of strong working relationships. Joint histories have been created as partners share power in coming to decisions and then acting on them.

The engagement with communities and physicians in Northern BC is only partially reflected by the literature that is increasingly calling for patient or physician engagement in the health system’s transformation (Best *et al.*, 2012). Instead of involving or engaging patients and physicians in a health system’s endeavor, NH has been actively seeking to become a good partner. Northern Health sometimes, but not always, takes the lead. The shared vision of improved health and improved services within a healthy community has meant that action on the part of the partners happens separately and together. The partners consistently try to bring tensions to the surface, acknowledge them, and work them out. Initiatives are not always within the purview of NH; power and authority are by necessity, shared.

While it is commonly held that “explicit alignment of formal vision and goals” (Best *et al.*, 2012, p. 436) and communication is important, this study has shown the critical importance of a provincially supported regional strategic plan created created jointly by partners and with constant attention to language over time. Banning phrases such as “rolling out” or “driving out,” and continued attention to the language of change and of action (“we,” “our,” vs “they”) over the years is notable. The attention to language communicates inclusivity and respect for local needs, local capacity, and local initiatives. While the importance of language has received attention in research on iterative approaches to organizational change (Reay *et al.*, 2013) the explicit attention to language has yet to be fully explored in research on whole system change.

Flexibility has characterized the approach to change in Northern BC. Within the overall goal and direction, being strategic yet opportunistic has given room to experiment at both regional and local levels. Having the room to try new things, and using that room to make a difference is necessary to enact primary healthcare reform (Reay *et al.*, 2017). Northern Health leaders have had to engage in a delicate balance of encouraging, cautioning, molding, and being open to change while maintaining regional accountability and avoiding unnecessary duplication. Importantly, regional leaders have attempted to monitor but not shut down local initiatives that address the goals of the transformation. This has not always been easy or successful. With so much of the drive for health system transformation originating from the center, either provincially or regionally, the commitment among the partners to the people in their communities has been evident. Maintaining this commitment in the face of ongoing political forces (Hunter *et al.*, 2015) will continue to prove challenging.

The principles and rules identified by writers on large system transformation (e.g. Best *et al.*, 2012; Greenhalgh and Papoutsi, 2018) were seen in part in this study, as were elements of process models like Pettigrew’s receptive context model as identified by Hunter *et al.* (2015) and in Suter *et al.*’s (2009) principles. Best *et al.*’s (2012) description of large system transformation as “interventions aimed at coordinated, system-wide change affecting multiple organizations and care providers, with the goal of significant improvements in the efficiency of healthcare delivery, the quality of patient care, and population-level patient outcomes” (p. 422) suggests a level of coordination that fails to capture the various ways in which the small communities took actions to transform primary healthcare so that it could work over time within their communities. The principles and rules were also insufficient to depict the ebbs and flows of the iterative change processes. They could not capture the ongoing interplay of the context, including geographical context, and the political and social dynamics that contributed to the timing, and the advancement or delay of transformation.

Engaging in such a large, multifaceted change process comes with multiple challenges. Such processes are imbued with tensions, including tensions between professionals, tensions in goals, tensions related to power, tensions about timing and pacing of change, and tensions inherent in making local changes within a large, complex, publicly accountable regional health system that needs to accomplish provincial mandates (Hanlon *et al.*, 2019a, b; Snadden *et al.*, 2019) Instead of seeking to manage or to create a synthesis of tensions when they are managed (Van de Ven, 1992), NH leaders have understood tension as described by McCormack *et al.* (2013) as:

[…] a strain that generates differences amongst partners; presenting an opportunity for partners to engage in reciprocal learning […] [where] partners are challenged to think differently and to move […] to a new space where creative and effective solutions are found.

(p. 18)

Northern Health and its partners have found ways to embrace the tensions, and work with them to create a solid foundation for long-term organizational and health system change.

Through purposeful, respectful attempts to understand their partners, NH leaders have sought to dwell within each side of the various tensions at different points in time and in different contexts. In this way, NH leaders have come to understand the perspectives and realities of those experiencing the tensions. They have learned how there can be both certainty and uncertainty, both rapid change and slow change, both directive change and organic change, both provincial directions and regional and local flexibility. Through experiencing the tensions while being anchored in their relationships with their partners, guided by the clear end-in-view of improving the health of northerners in healthy communities, the partners in this change process have found, and continue to find, ways to take calculated risks, withstand setbacks, and work toward long-term goals.

### Strengths and limitations

Undertaking a study within one sparsely populated region offers both strengths and limitations. It is a strength that NH, northern communities, and northern physicians could be studied over time; the small populations in each community meant that the key informants could speak of the experiences of many in their communities and organizations. Few changes in membership were experienced among the NH Executive and Board leadership. Sufficient numbers of municipal leaders and physicians agreed to interviews over the study period, allowing the study of change over time. Over the next three years, the continuing partnering for change research will capture the evolving collaboration among NH, physicians, municipalities and First Nations in primary healthcare transformation. Although it provides a rich description in a particular context, the study approach does not lead to generalization. It is not known how transferable the findings are to other geographic settings. Confirmation of the findings by the participants was not sought, but the findings and interpretations resonated with many different audiences over the course of the study.

## Conclusion

In sum, Northern Health, northern physicians, and communities continue to be in the process of transforming the system of care as an outcome of re-orienting health services to focus on the health of the population and patient-centered care. Instead of creating new work-arounds and adding layers of services, NH, with its partners, has been altering primary healthcare by focusing on patients, families and communities.

This long-term approach to change has the potential to create a new system, rather than rearranging components of a broken system. In Northern BC, the process is still underway and the outcomes are not yet fully realized. A critical component is the respectful partnering approach that can create a culturally safe health delivery system. Northern Health, FNHA, and First Nations communities are engaging in the creation of a shared vision that respects different ways of knowing. Given the work to date in transforming primary healthcare, there is a strong potential to improve the lives and health of those living in rural and northern communities.

This case study provides lessons of how to engage locally, create spaces for dialogue to acknowledge tensions, and put in place meaningful processes by which partners are enabled to co-create a long-term vision leading to action for healthcare sustainability. Perhaps more importantly, however, this case highlights the strength of vision, commitment, and opportunism needed to bring about system change in the context of “real world” messiness that is so characteristic of healthcare. As such, the findings are not so much a blueprint for how to do things, as they are a way of recognizing patient-focused whole system change as a journey with a destination in sight, but whose route will be altered by both hazard and opportunity.

## Figures and Tables

**Table I:** Regional and community interviews

	Year 1	Year 2	Year 3
NH board and executive	11	11	12
NH administrators and directors	9	15	17
Regional interviews total	20	26	29
NH staff, managers and care providers	34	31	32
Physicians	10	5	6
Community organizations/municipal leaders	16	15	15
Seven communities – Interviews total	60	51	47
Overall total	80	77	82

## Appendix

### Partnering for change

#### Interview guide

1. As you know, this research project has been created to study the processes of Primary Healthcare transformation. Can you tell me about how you first became involved in this process of PHC transformation and how it has developed over time?
2. Taking advantage of opportunities is something that we see in times of change. Can you think of an opportunity that presented itself, how you recognized it and what led to you or the organization being able to respond to it?
3. What tensions have you had to work through so far in this process of change?
  - Can you think of any turning points where tensions were created or resolved?
  - What came into those situations to move things along?
4. From your perspective, what relationships between organizations have been needed to make the changes in providing primary health care?
  - Probes: How have people worked across organizations to make the changes in PHC?
5. How have particular individuals contributed to relationship building? Who are these individuals? What are the special things that they do to make things happen?
  - Probes:
    What roles have people taken on that contributed to an effective partnership? Have individuals and roles changed over time?
    How have new individuals/organizations been added to your network?
    What has happened to the partnership and the planning/implementation processes when individuals have joined or left a network?
6. In general, what has helped to build trust within your current relationships with community and professional partners? What kinds of local and non-local support have been required for the network to succeed?
7. There are times when otherwise effective partnerships and networks do not run well. In your experience, what factors do you think have led to ineffectiveness in networking or networks?
8. What would you say is working better now due to the networking and partnering?
  - What innovation has occurred? Has this created value?
9. What changes do you see in the ways that primary health care services are provided? Do you think that this is on the right track? What do you see as next steps?
  - Probes:
    What are the main benefits/challenges to you, your work/practice, PHC patients, the community of developing/maintaining these networks and relationships? Do these benefits/challenges extend beyond the particular initiative?
10. Sometimes in a process of change you look back and all of a sudden you realize that there have been changes. Do you remember any situations like that? What led up to that point? Where did the changes go after that?
11. When we come back next year to interview you – what do you predict we will see?
12. Is there anything I should have asked you, but have not?
